# Bioactive metal oxide nanoparticles from some common fruit wastes and *Euphorbia condylocarpa* plant

**DOI:** 10.1002/fsn3.1853

**Published:** 2020-08-31

**Authors:** Ghodrat Mahmoudi, Ebrahim Sufimahmoudi, S. Mohammad Sajadi

**Affiliations:** ^1^ Department of Chemistry Faculty of Science University of Maragheh Maragheh Iran; ^2^ Department of Nutrition College of Health Technology Cihan University‐Erbil Kurdistan Region Iraq; ^3^ Department of Phytochemistry SRC Soran University Soran Iraq

**Keywords:** biological activities, *Euphorbia condylocarpa*, fruit wastes, green nanoparticles

## Abstract

For the first time, the potential of orange and banana peels as fruit wastes was evaluated in contrast with *Euphorbia condylocarpa* as a widely distributed medicinal plant of Kurdistan, Iran, for biosynthesis of Fe_3_O_4_, CuO, ZnO, and TiO_2_ NPs. The extracts of the green sources were assessed to monitor the bioreducing phytochemicals inside them using the UV‐Vis spectrophotometer. Moreover, the obtained green nanoparticles were identified using the micrograph and diffractogram techniques to show their size, shape, and morphology. Also, the antibacterial activities of the green NPs were investigated against common pathogenic bacteria of *Pseudomonas aureus*, *Staphylococcus aureus*, *Escherichia coli*, and *Klebsiella pneumoniae*.

## Graphical abstract

The potential of orange and banana peels as fruit wastes was evaluated in contrast with Euphorbia condylocarpa for biosynthesis of Fe_3_O_4_, CuO, ZnO, and TiO_2_ NPs. The extracts of the green sources were assessed to monitor the bioreducing phytochemicals inside them using the Uv‐Vis spectrophotometer. Moreover, the obtained green nanoparticles were identified using the micrograph and diffractogram techniques to show their size, shape, and morphology. Also, the antibacterial activities of the green NPs were investigated against common pathogenic bacteria of *Pseudomonas aureus*, *Staphylococcus aureus*, *Escherichia coli*, and *Klebsiella pneumoniae*.

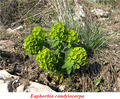

## 1 INTRODUCTION

Novel strategies are required for the achievement of safe and effective new and clean technologies, moving toward green technology development that significantly contributes to environmental sustainability through the production of nanostructures without causing harm to human health or the environment (Soni, Mehta, Soni, & Goswami, 2018; Velusamy, Venkat Kumar, Jeyanthi, Das, & Pachaiappan, 2016; Verma, Gautam, Bansal, Prabhakar, & Rosenholm, 2019). The term of nanotechnology defines the process of designing, synthesis, and application of any particle having size 1–100 nm which introduce a spectrum of nanoscale materials with enhancement of solubility and bioavailability, improvement of stability, suppression of toxicity, improvement of pharmacological activity, sustained delivery, improving tissue macrophage circulation, and defense against physical and chemical degradation. Nowadays, biosynthesis of nanoparticles attracts so much attention due to their controllable size, shape, and surface properties and also development demand of environmental friendly, nontoxic, and cheap technology for material synthesis (Abdelghany et al., 2018; Chartarrayawadee et al., 2020). A number of green and naturally occurring sources such as microorganisms including bacteria, fungi, yeasts, algae, and plants either intra‐ or extracellular have enough capability to synthesis of antibacterial, antifungal, antiviral, antioxidant, and anticancer nanoparticles (Rafique, Sadaf, Shahid Rafique, & Bilal Tahir, 2017).

Global demand for foods with additional health benefits for delivering valuable phytochemicals through different foods has increased over the last decades. Despite the foods, food waste generated during the preparation of meals and any foods that are not consumed are rich sources of phytochemicals with several types of biological activities. Every day, large quantities of foods almost including one third of the food produced in the world for human consumption wasted and then caused a serious environmental and economic problem due to their disposal. However, food wastes are rich and inexpensive source of beneficial phytochemicals, and their exploitation for recovery of these compounds and utilization as functional additives in different products represent a challenging field for researchers (Kumar, Nath Yadav, Kumar, Vyas, & Singh Dhaliwal, 2017; Thakur, Kumar Biswas, & Singh, 2017; Tiwari, Brunton, & Brennan, 2013).

Medicinal effects of fruit as the simplest form of functional foods and their extracts obtained from fresh tissues, residual parts, and wastes caused to their application in various sectors, like cosmetics, pharmaceuticals, and food industries due to high amounts of bioactive compounds (Baiano, 2014; Drosou, Kyriakopoulou, Bimpilas, Tsimogiannis, & Krokida, 2015; Filip, Pavlić, Vidović, Vladić, & Zeković, 2017). In fact, fruits are valuable source of secondary metabolites with a wide range of applications which their intake helps in overall health improvement. Residues consist of fruit skin/peels, sugarcane bagasse, residual part, and seeds are among the major sources of municipal solid waste and bioactive phytochemicals rich in antioxidant compounds apart from dietary fiber, minerals, phenolic compounds, carotenoids, tannins, vitamin C, and dietary fiber. In some cases, analysis of fruit wastes revealed that the total polyphenol and flavonoid contents are much higher in the fruit wastes from peel and seeds as compared to the edible part of fruit of mangoes, lemons, oranges, and grapes. There is growing interest of consumers toward citrus fruit antioxidant‐rich compounds (e.g., ascorbic acid) and essential oils for their effects on human health promotion and disease risk reduction (Nobre, Palavra, Pessoa, & Mendes, 2009; Šeregelj et al., 2018).

The *Euphorbiaceae* (spurge) are a large family of flowering plants observed in tropical and nontropical areas as herbs, shrubs, or trees. In most cases, a milky and poisonous latex is a characteristic of the plants of this family. The plants of these family are a very rich source of bioactive phytochemicals such as terpenoids, flavonoids, alkaloids, tannins, phenolic acids, and porphyrins. In addition of the rich phytochemical content of this family, their latex is used as a laxative in folk medicine (Maryami, Nasrollahzadeh, Mehdipour, & Sajadi, 2017; Nasrollahzadeh, Atarod, & Sajadi, 2017; Sajadi, Nasrollahzadeh, & Maham, 2016). The huge variety of phytochemical content of the family revealed its effective remedy for many diseases like antidiarrhea, antioxidant, antibacterial, antidiabetic, and anti‐inflammation diseases (Jassbi et al., 2006; Rizk, 1987). During this study, we employed the *Euphorbia condylocarpa* aqueous extract as reducing and stabilizing agent for phytosynthesis of some metal oxide nanoparticles and investigation of their bioactivity characteristics with the same nanostructures synthesized by using some common fruit wastes.

## 2 MATERIALS AND METHODS

### 2.1 Instrumentations

All used chemicals were of Merck purity. XRD analysis was carried out using Panalytical X'Pert^3^ Powder using Cu Kα radiation equipped with a diffractometer system X'Pert Pro. The diffraction pattern was recorded for 2θ from 5 to 80° and a 2θ step scan of 0.010° was used, counting for 0.5 s at every step. The voltage and current of the generator were set at 45 kV and 40 mA, respectively. The prepared nanomaterials coated with gold using coater machine, then particles morphology was investigated using FEI (Quanta 450) scanning electron microscopy equipped with Quantax EDS—XFlash 6/10 microanalyzer for detecting chemical composition of the prepared nanomaterials. UV‐visible spectral analysis was recorded on a double‐beam spectrophotometer (Super Aquarius) to monitor the antioxidant extracts of the employed green sources.

### 2.2 Fruit waste and plant material

**Figure 1:**
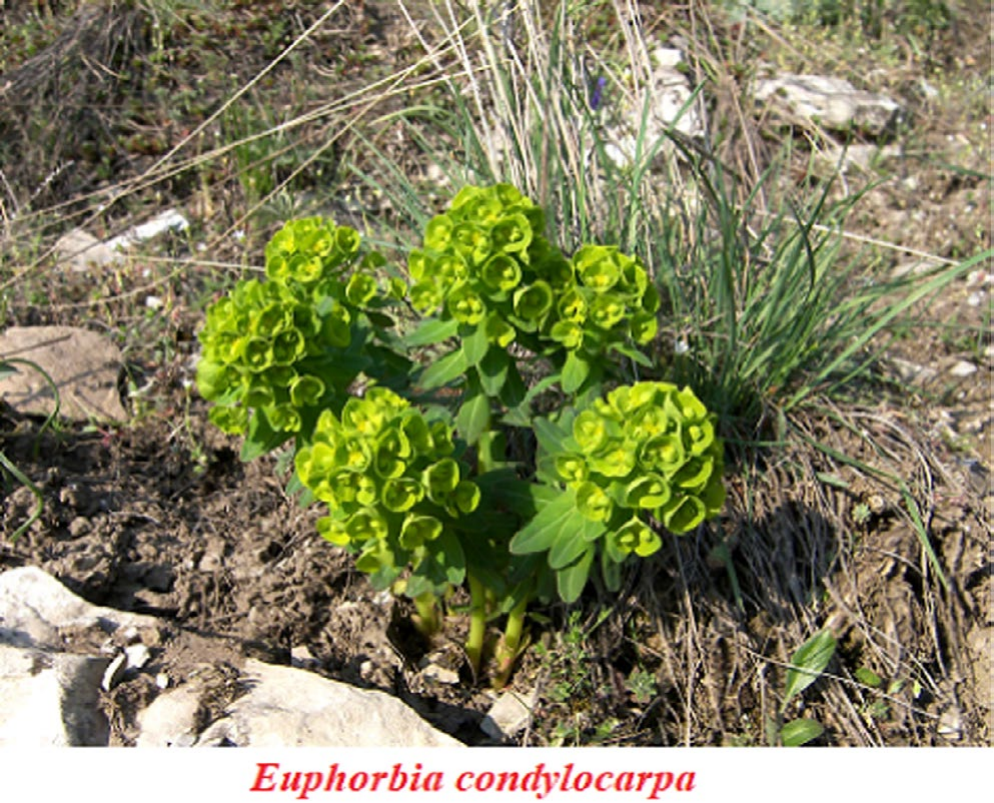
The image of *E. condylocarpa* plant

The whole *E. condylocarpa* plant was collected from Sarshive Region in Saqqez city of Iranian Kurdistan, Figure 1. Fruit wastes were collected from the residual of wasted parts of fruits after daily consumption.

### 2.3 Preparation of extracts

20 g root powders of *E. condylocarpa* was mixed to 100 ml distilled water at 85°C for 30 min while stirring on magnetite heater. Then, the mixture was centrifuged at 7,000 rpm and supernatant separated as extract. In case of obtaining the extract of fruit wastes including banana and orange peels, the procedure was the same.

### 2.4 Green synthesis of Fe_3_O_4_, CuO, ZnO, and TiO_2_ NPs

**Scheme 1:**
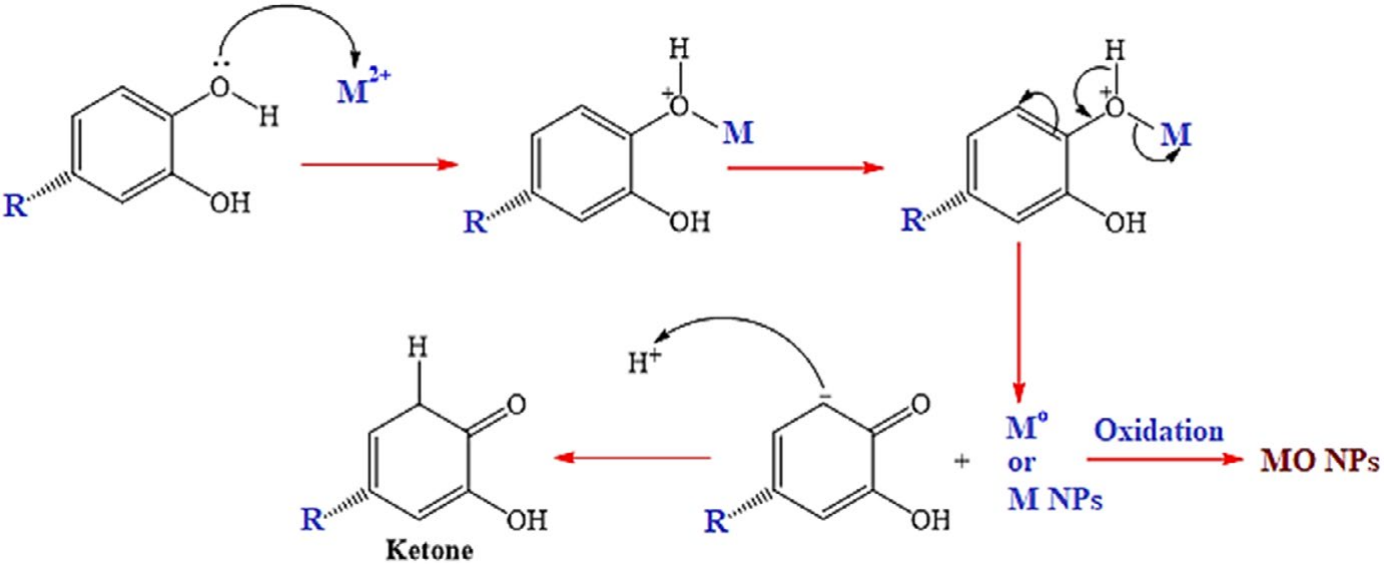
Possible mechanism for biosynthesis of MO NPs using the antioxidant content of the plant and fruit wastes

0.5 g FeCl_2_ and 1 g FeCl_3_ (in case of CuO, ZnO, and TiO_2_ NPs, the amount of salts was 0.5 g CuCl_2_.2H_2_O, 0.8 g ZnCl_2,_ and 0.5 g TiO(OH)_2_) were mixed with 20 ml *E. condylocarpa* extract (in case of banana and orange peel wastes, the amount of employed extract was the same) at pH 9 (as adjusted using Na_2_CO_3_) while stirring at 80 ºC for 5 hr (in case of CuO, ZnO, and TiO_2_, the time was 2, 1.5, and 2.5 hr, respectively) as the completion time of the reaction under reflux conditions. Of course, the time of changing the color of the mixture for each nanoparticle originated from different green sources demonstrating the SPR (surface plasmon resonance) signals and starting the formation of nanoparticles is different. The obtained precipitates from each case were separated using filtration, washed with 60% hydroethanolic solution to remove the impurities, and then dried and kept to further investigations, Scheme 1.

### 2.5 Anti‐bacterial activity of green‐synthesized nanoparticles

The disk diffusion method was employed to assess the antipathogenic bacterial activity of green‐synthesized nanoparticles from fruit wastes and *E. condylocarpa* against common pathogenic bacteria of *Pseudomonas aureus*, *Staphylococcus aureus*, *Escherichia coli*, and *Klebsiella pneumoniae* and chloramphenicol as positive control. The bacterial suspensions were inoculated at 10^5^ CFU/ml of Mueller–Hinton agar. At the same conditions, sterile filter paper disks (6 mm D) loaded separately with green nanomaterials (100 µl) and chloramphenicol on the top of Mueller–Hinton agar plates and then incubated at 37°C for 24 hr. The diameter of inhibition zone was measured after 24 hr of incubation using a caliper and tabulated in mm.

## 3 RESULTS AND DISCUSSION

Plants and majority of edible fruits as biofactories contain a diverse type of bioactive secondary metabolites. Besides them, it has been revealed that fruit wastes are precise sources of phytochemicals as which the total polyphenol and flavonoid contents of some fruit wastes are higher than the consumable parts of the fruit. Therefore, in this study we focus on using the banana and orange wastes potential for synthesis of bioactive nanoparticles and comparison of them with the same nanostructures produced by powerful antioxidant extract of *Euphorbia condylocarpa* plant.

### 3.1 UV‐visible monitoring of the aqueous extracts from E. condylocarpa plant, banana, and orange peels

**Figure 2:**
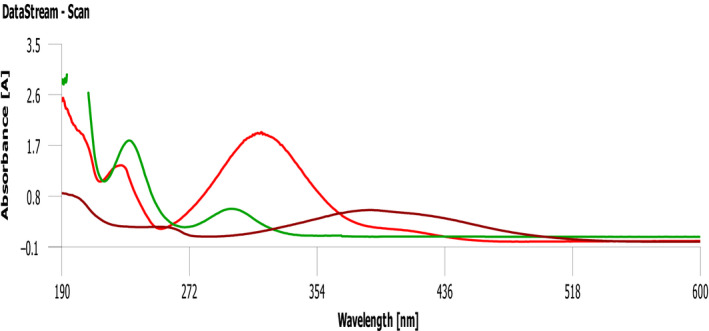
UV‐Vis spectra of aq. extracts of *E. condylocarpa* root (red), orange peel (green), and banana peel (brown)

The UV‐Vis spectra of each aqueous extracts of *E. condylocarpa* plant and banana and orange peels as real samples of fruit wastes show the potential of these extracts for biosynthesis of nanoparticles as they have signals at the range of 220–270 nm and also 315–370 nm assigned to the benzoyl and cinnamoyl rings of phenolic antioxidants, respectively. Although according to the UV‐vis spectra of the samples, the intensity of the plant and orange extract signals probably indicates the higher concentration of these antioxidant systems in their extracts. Generally, based on this analysis they are very good bioreducing sources to green synthesis of nanoparticles, Figure 2.

### 3.2 Identification of the green‐synthesized nanoparticles

**Figure 3:**
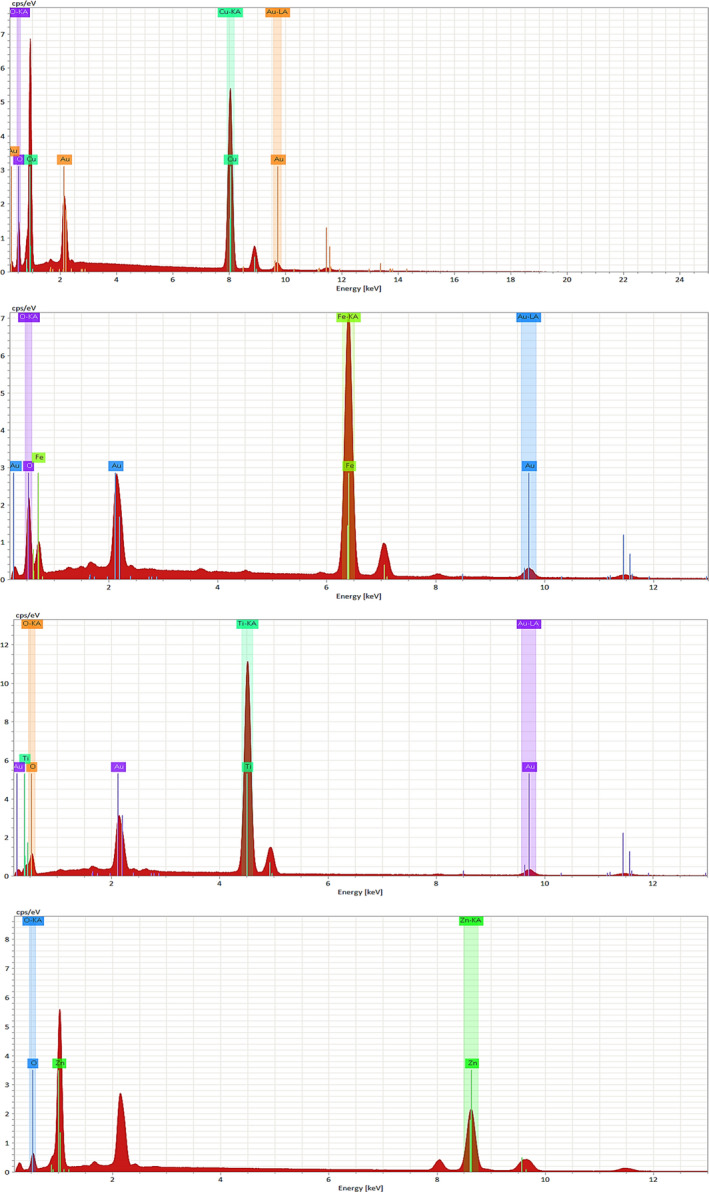
The EDS analysis of green‐synthesized *nanoparticles using fruit wastes and E. condylocarpa*

The green‐synthesized nanostructures using *E. condylocarpa* plant and also orange and banana fruit wastes were identified using the micrograph (*SEM* and EDS) and X‐ray diffraction techniques. The EDS spectrum for all nanoparticles from mentioned sources demonstrated their related elements of Cu and O (for CuO NPs), Fe and O (for Fe_3_O_4_ NPs), Zn and O (for ZnO NPs), and Ti and O (for TiO_2_ NPs) as which besides their green sources, each nanoparticle has a very suitable purity and almost the same similarity according the EDS analysis, Figure 3.

**Figure 4:**
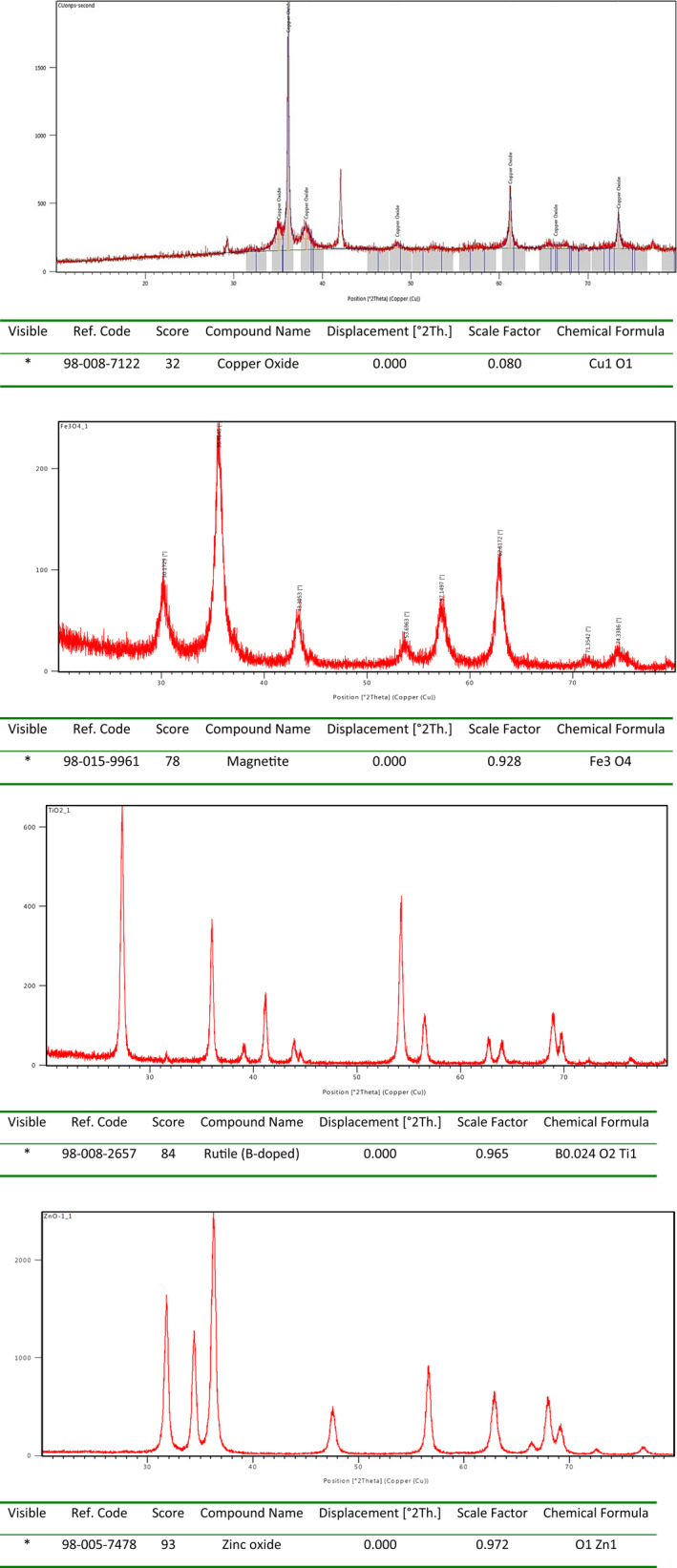
The XRD diffractogram of green‐synthesized nanoparticles using fruit wastes and *E. condylocarpa*

Furthermore, the X‐ray diffraction was employed to investigate the phase purity and crystallinity of green‐synthesized nanostructures. The XRD diffractograms of all green‐synthesized nanoparticles show the synthesis of pure crystalline nanoparticles with a homogeneous morphology. The intensity of crystalline signals in all diffractograms concerning the same nanoparticles was approximately the same which visibly depicted the same ability of green sources in production of crystalline nanostructures, Figure 4.

**Figure 5:**
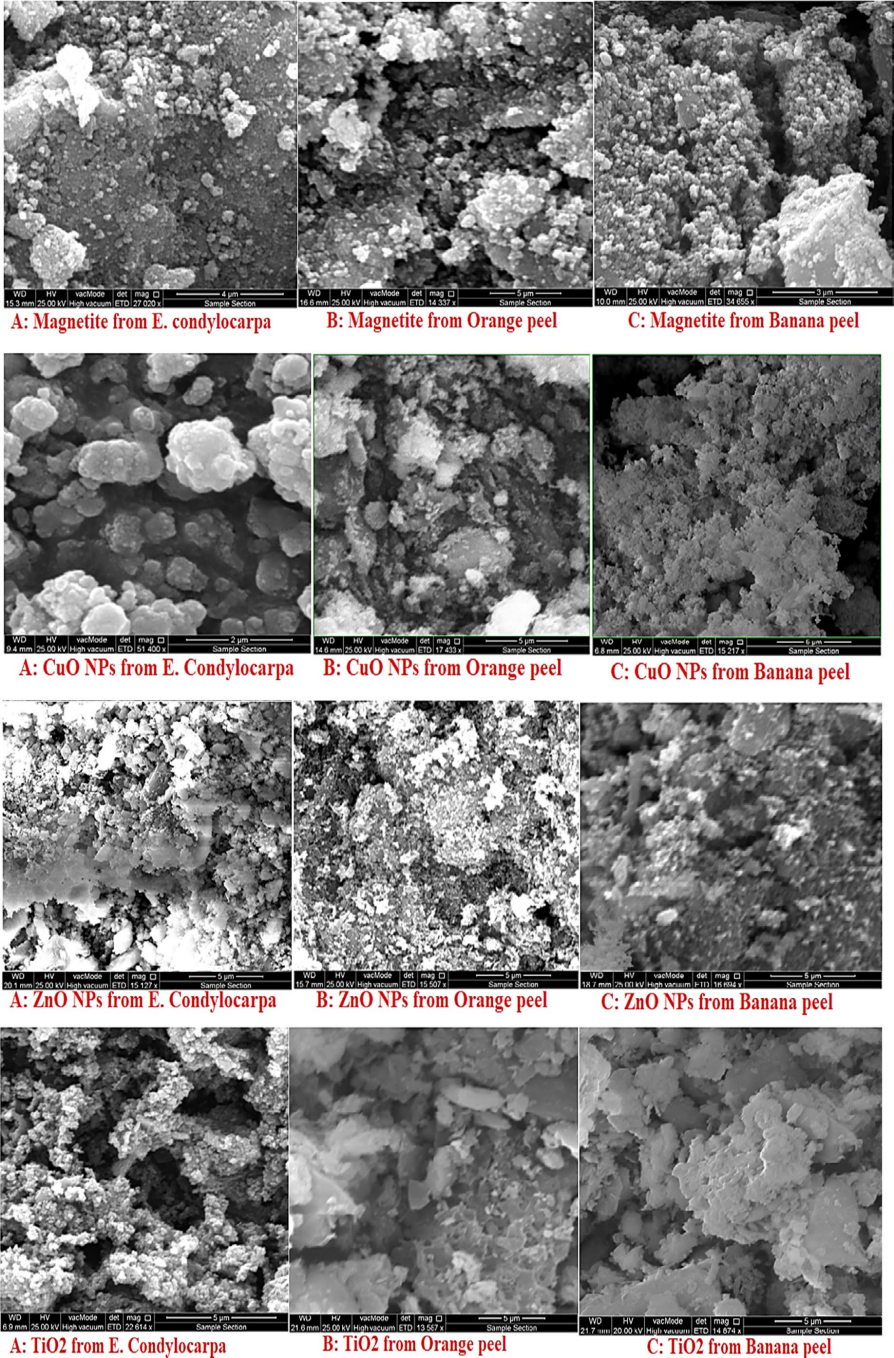
*SEM* analysis of various metal oxide nanoparticles using the antioxidant and reducing ability of different green sources including plant and fruit wastes

The scanning electron microscopy (*SEM*) micrograph revealed the important properties of nanoparticles such as size, shape, morphology, and homogeneity, Figure 5. In case of magnetite NPs, beside more agglomeration of green‐synthesized NPs using banana source, the spherical‐shaped nanoparticles synthesized using all sources have approximately the same size ranging 30–85 nm and homogenous morphology. The scanning electron microscopy analysis of CuO NPs demonstrated more varieties from the view of the size, shape, and morphology of NPs as strongly refers to the type of green sources. The CuO NPs synthesized using *E. condylocarpa* and orange peel shows spherical shape and almost homogeneous morphology while in case of banana peel, despite the smaller size of CuO NPs, they show no distinguished shape and morphology. Also, the amount of agglomeration for CuO NPs originated from banana peel extract is more than the *E. condylocarpa* plant and orange peel. Furthermore, the semispherical shape of ZnO NPs synthesized from all sources has almost the same size and morphology with a considerable amount of agglomeration. Despite the high amount of homogeneity in ZnO NPs, there are a lot of differences between the homogeneity of TiO_2_ NPs. According the Figure 5, the titanium dioxide nanoparticles obtained from the extract of *E. condylocarpa* have the network morphology and spherical shape with less size than the same nanostructures obtained by fruit waste sources. Although the size of TiO_2_ NPs from the fruit wastes is greater, their planar forms and distinguished diversity in surface homogeneity especially in case of the nanoparticles synthesized by orange peel extract distinguished them from the NPs obtained by the plant extract (Table 1).

**Table 1:** Characteristics of the green‐synthesized NPs from *E. condylocarpa* plant and fruit wastes

Green source	Nanoparticle	Size	Shape	Morphology	Surface homogeneity	Agglomeration	Reaction Time	Formation Time (SPR time)
*Euphorbia condylocarpa*	Fe_3_O_4_ NPs	30–85 nm	Spherical	Granular	Homogenous	A few	5 hr	15 min
*E. condylocarpa*	CuO NPs	70–100 nm	Spherical	Granular	Homogenous	A few	2 hr	25 min
*E. condylocarpa*	ZnO NPs	50–80 nm	Semispherical	Granular	Homogenous	A lot	1.5 hr	16 min
*E. condylocarpa*	TiO_2_ NPs	40–60 nm	Spherical	Network	Homogenous	A few	2.5 hr	41 min
Orange peel	Fe_3_O_4_ NPs	30–85 nm	Spherical	Granular	Homogenous	A few	5 hr	25 min
Orange peel	CuO NPs	30–60 nm	Spherical	Granular	Homogenous	A few	2 hr	17 min
Orange peel	ZnO NPs	50–80 nm	Semispherical	Granular	Homogenous	A lot	1.5 hr	20 min
Orange peel	TiO_2_ NPs	≥ 100 nm	Sheet form	Planar	Heterogeneous	A few	2.5 hr	45 min
Banana peel	Fe_3_O_4_ NPs	30–85 nm	Spherical	Granular	Homogenous	A few	5 hr	34 min
Banana peel	CuO NPs	20–40 nm	Not clear	Granular	Heterogeneous	A lot	2 hr	27 min
Banana peel	ZnO NPs	50–80 nm	Semispherical	Granular	Homogenous	A lot	1.5 hr	22 min
Banana peel	TiO_2_ NPs	≥ 100 nm	Semispherical	Planar	Heterogeneous	A lot	2.5 hr	83 min

### 3.3 Antimicrobial activity

**Figure 6:**
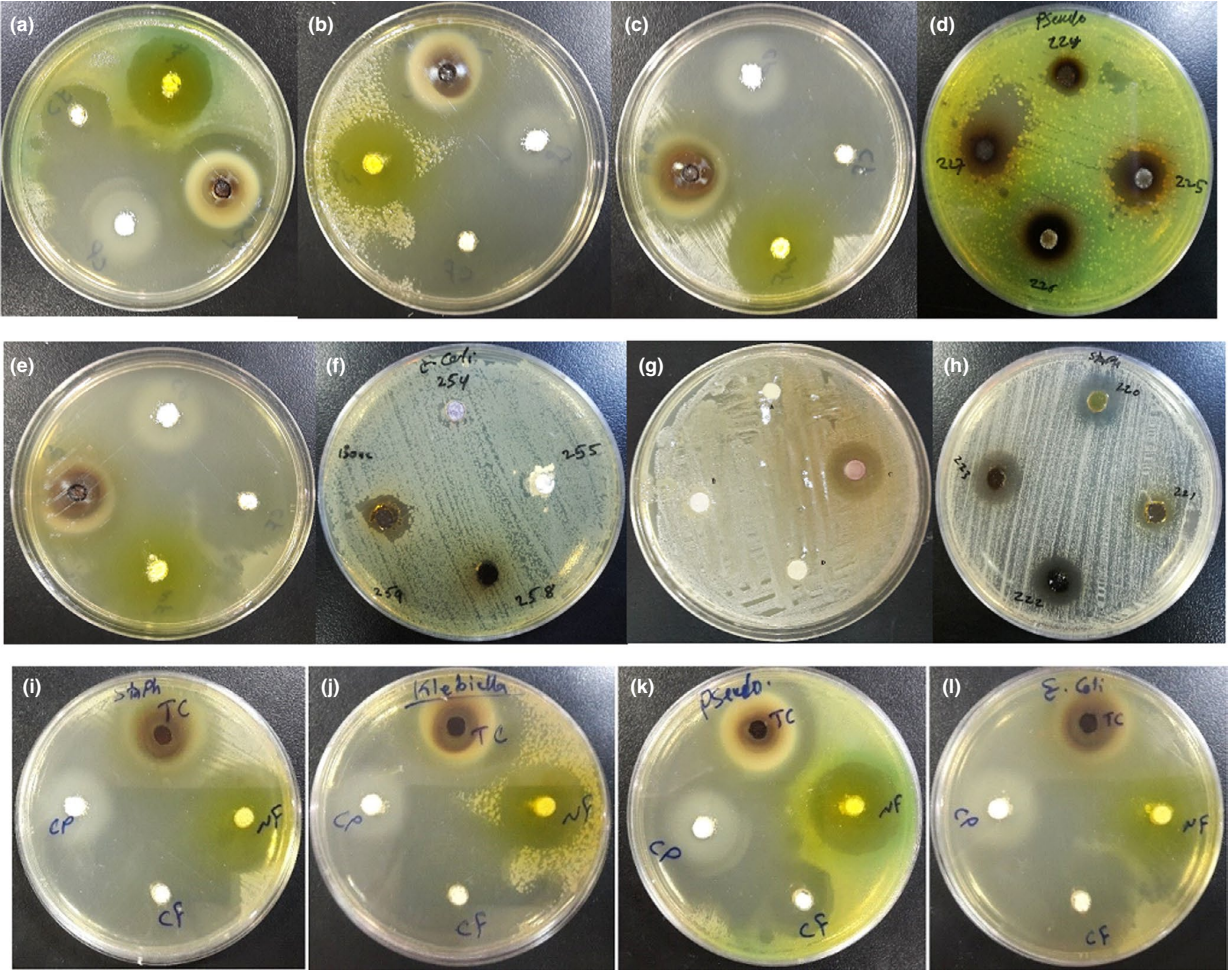
The inhibition zone of green nanoparticles synthesized by *E. condylocarpa* (*A: S. aureus*; *B: E. coli*; *C: K. pneumonia*; D: *P. aureus*), orange peel (*E: P. aureus*; *F: E. coli*; *G: K. pneumonia*; *H: S. aureus*), and banana peel (*I: S. aureus*; *J: K. pneumonia*; *K: P. aureus*; *L: E. coli*) sources

The antibacterial activity of green‐synthesized NPs was subjected to test with common pathogenic bacteria of *Pseudomonas aureus*, *Staphylococcus aureus*, *Escherichia coli*, and *Klebsiella pneumoniae*. The chloramphenicol was used as positive control to carefully evaluate the antibacterial activities. The inhibition zone for all types of nanoparticles fabricated by *E. condylocarpa*, orange peel, and banana peel sources were shown in Figure 6. The inhibition zone diameters of all samples are recorded in Table 2.

**Table 2:** The inhibition zone of green‐synthesized NPs using various green sources against common pathogenic bacteria

Green source	Nanoparticle	*Pseudomonas aureus* (mm)	*Staphylococcus aureus* (mm)	*Escherichia coli* (mm)	*Klebsiella pneumonia* (mm)
*E. condylocarpa*	Fe_3_O_4_ NPs	28 mm (No. 227)	44 mm (Tc)	46 mm (Tc)	42 mm (Tc)
*E. condylocarpa*	CuO NPs	18 mm (No. 226)	24 mm (Cf)	18 mm (Cf)	19 mm (Cf)
*E. condylocarpa*	ZnO NPs	11 mm (No. 224)	42 mm (Cp)	36 mm (Cp)	10 mm (Cp)
*E. condylocarpa*	TiO_2_ NPs	25 mm (No. 225)	39 mm (Nf)	35 mm (Nf)	42 mm (Nf)
Orange peel	Fe3O4 NPs	21 mm (Tc)	13 mm (No. 223)	16 mm (No. 259)	24 mm (C)
Orange peel	CuO NPs	41 mm (Nf)	14 mm (No. 222)	11 mm (No. 258)	No response (B)
Orange peel	ZnO NPs	17 mm (Cp)	13 mm (No. 220)	No response (No. 255)	No response (A)
Orange peel	TiO_2_ NPs	39 mm (Cf)	10 mm (No. 221)	No response (No. 254)	No response (D)
Banana peel	Fe_3_O_4_ NPs	44 mm (Tc)	40 mm (Tc)	41 mm (Tc)	42 mm (Tc)
Banana peel	CuO NPs	32 mm (Cf)	No response (Cf)	43 mm (Cf)	No response (Cf)
Banana peel	ZnO NPs	18 mm (Cp)	18 mm (Cp)	18 mm (Cp)	18 mm (Cp)
Banana peel	TiO_2_ NPs	45 mm (Nf)	47 mm (Nf)	46 mm (Nf)	46 mm (Nf)

The antibacterial activities of green‐synthesized NPs against some of the most common pathogenic bacteria revealed a potent bioactivity for most of them. Among the green NPs, those synthesized by *E. condylocarpa* as a green source generally showed more antibacterial activity than the NPs fabricated by the orange and banana peels. Also, for the TiO_2_ NPs synthesized by banana peel, the inhibition zones (mm) for all types of bacteria are even more than chloramphenicol as positive control (38 mm). In case of orange peel, some NPs showed no response against *E. coli* (ZnO NPs and TiO_2_ NPs) and *K. peneumonia* (CuO NPs, ZnO NPs and TiO_2_ NPs); furthermore, the same result is seen for CuO NPs fabricated by banana source about *S. aureus* and *K. peneumonia* bacteria (Table 2). Beside the size shape, morphology, and surface area of the nanoparticles indicated in Table 1, the antibacterial activity of green NPs probably refers to the accumulation of various bioactive phytochemicals on the surface of NPs. In fact, due to the large surface area of nanoparticles, a high concentration of phytoconstituents (hyperaccumulation of bioactive phytochemicals) adsorbed on the surface of NPs led to their strong bioactivity and long‐term stabilization against decomposition and deformation reactions.

## 4 CONCLUSIONS

In this study, the ability of *Euphorbia condylocarpa* plant, and orange and banana peels as fruit wastes and reducing agents were examined to biosynthesis of a series of the same nanoparticles of Fe_3_O_4_, ZnO, TiO_2,_ and CuO NPs through an eco‐friendly, economic, and rapid method. The structure of all green‐synthesized NPs using micrograph and diffractogram techniques demonstrated their absolute fabrication using the green sources. Also, the green‐synthesized NPs were tested against *Pseudomonas aureus*, *Staphylococcus aureus*, *Escherichia coli*, and *Klebsiella pneumoniae* as common pathogenic bacteria which revealed a very good bioactivity due to the hyperaccumulation of bioactive phytochemicals on the surface of them. Furthermore, the structure and antibacterial monitoring of all green‐synthesized NPs demonstrated that the plant source of *Euphorbia condylocarpa* has more potential than the fruit wastes to biosynthesis of more bioactive nanoparticles.

## CONFLICT OF INTEREST

The authors declare that they do not have any conflict of interest.

## ETHICAL APPROVAL

This study does not involve any human or animal testing.
